# Development of portable sensor for the detection of bacteria: effect of gold nanoparticle size, effective surface area, and interparticle spacing upon sensing interface

**DOI:** 10.1186/s11671-023-03826-4

**Published:** 2023-03-18

**Authors:** Khadija Al-Yahmadi, Htet Htet Kyaw, Myo Tay Zar Myint, Rahma Al-Mamari, Sergey Dobretsov, Mohammed Al-Abri

**Affiliations:** 1 Nanotechnology Research Center, Sultan Qaboos University, Al-Khoud, P.O. Box 33, 123 Muscat, Oman; 2 Department of Physics, College of Science, Sultan Qaboos University, Al-Khoud, P.O. Box 36, 123 Muscat, Oman; 3 UNESCO Chair. Department of Marine Science and Fisheries, College of Agricultural & Marine Sciences, Sultan Qaboos University, Al-Khoud, P.O. Box 34, 123 Muscat, Oman; 4 Department of Petroleum and Chemical Engineering, College of Engineering, Sultan Qaboos University, Al-Khoud, P.O. Box 33, 123 Muscat, Oman

**Keywords:** Gold nanoparticle, Chitosan, Three electrodes sensor, Bacteria detection, Detection limit

## Abstract

In this study, systematic development of a portable sensor for the rapid detection of *Escherichia coli* (*E. coli*) and *Exiguobacterium aurantiacum* (*E. aurantiacum*) was reported. A conductive glass was utilized as a substrate and developed the electrode patterns on it. Trisodium citrate (TSC) and chitosan-stabilized gold nanoparticles (AuNPs) (CHI-AuNP-TSC) and chitosan-stabilized AuNPs (CHI-AuNP) were synthesized and utilized as a sensing interface. The morphology, crystallinity, optical properties, chemical structures, and surface properties of immobilized AuNPs on the sensing electrodes were investigated. The sensing performance of the fabricated sensor was evaluated by using an electrochemical method to observe the current changes in cyclic voltammetric responses. The CHI-AuNP-TSC electrode has higher sensitivity toward *E. coli* than CHI-AuNP with a limit of detection (LOD) of 1.07 CFU/mL. TSC in the AuNPs synthesis process played a vital role in the particle size, the interparticle spacing, the sensor’s effective surface area, and the presence of CHI around AuNPs, thus enhancing the sensing performance. Moreover, post-analysis of the fabricated sensor surface exhibited the sensor stability and the interaction between bacteria and the sensor surface. The sensing results showed a promising potential for rapid detection using a portable sensor for various water and food-borne pathogenic diseases.

**Supplementary Information:**

The online version contains supplementary material available at 10.1186/s11671-023-03826-4.

## Introduction

Over the last decade, Au nanostructures have been utilized in various sensing applications such as the detection of water and food-borne pathogens, heavy metal ions, harmful gas molecules, and pesticides. [1–5]. Au nanostructures have unique properties, such as a large surface–to-volume ratio, high specific surface area, and excellent electrical and optical properties [6]. Colloidal-based synthesis is one of the most simple and cost-effective methods for synthesizing nanoparticles. The synthesis of metal ion precursors with various capping agents in organic and aqueous solutions has developed to achieve multiple sizes and shapes of Au nanostructures [7–9]. Owing to their bio-inertness and high physical and chemical stability, diverse applications such as colorimetric sensing, drug delivery, biological imaging, catalysis, and surface-enhanced Raman scattering probing used the colloidal form of Au nanoparticles (AuNPs) [10–14]. Substrate-bound Au nanostructures are more suitable for solid-state device fabrication owing to their stability and reusability. Au nanostructures can be immobilized on various substrates, including glass, conducting glass, fiber optics, silicon, and glassy carbon, and their properties differ from those of the colloidal form. Other alternative techniques, namely electrodeposition, electron beam evaporation, inert gas condensation, pulsed laser deposition, and ion-beam irradiation, have also been applied to fabricate Au nanostructures on desired substrates [15–19].

Although various techniques are used for AuNPs fabrication, colloidal-based synthesis is most commonly used because of its low cost, simplicity, ease of preparation, and unsophisticated surface functionalization. Different methods have been introduced to fabricate substrate-bound AuNPs, namely the self-assembly of colloidal AuNPs on functionalized substrates through electrostatic interaction [20, 21], Au precursor was loaded into diblock copolymer micelles to form ordered AuNPs arrays on the substrate [22], and layer-by-layer deposition of colloidal AuNPs on the substrate [23]. However, the immobilization of colloidal AuNPs on the desired substrate results in poor adhesion between the NPs and the substrate, which can cause the detachment of AuNPs from the substrate in different chemical environments. Thus, several techniques are applied to improve the physical stability of substrate-bound AuNPs. For instance, thermal treatment close to glass transition temperature enhances the adhesion between the NPs and the substrate for glass-substrate-bound AuNPs [24]. The electrochemical deposition of AuNPs on 4-phenyl-modified glassy carbon (GC) substrate achieved better attachment of AuNPs under sonication and electrochemical treatment [25]. Colloidal AuNPs mixed with chitosan solution and deposited on the substrate are a simple and alternative way to fabricate stable AuNPs-embedded polymer matrix nanocomposites [26].

Substrate-bound Au nanostructures are generally utilized as sensing surfaces in various types of water, food-borne pathogens, and heavy metal ions sensing applications [2, 5, 27]. Well-separated AuNPs immobilized on poly (styrene-*b*-4-vinyl pyridine) block copolymer-functionalized optical fibers significantly improve protein detection [28]. The label-free detection of bacteria was achieved with AuNPs dispersed on hafnium ditelluride nanosheets and utilized as a nanocomposite surface-enhanced Raman scattering (SERS) substrate [29]. Ochratoxin A aptamer functionalized Au nanorods surface was served as a localized surface plasmon resonance aptasensor chip for the detection of mycotoxin and ochratoxin A [30]. Another interesting paper-based sensing method was developed by immobilizing the antibody attached AuNPs to the bacteria detection zone together with Ag ions for signal enhancement and visualization [31]. AuNPs deposited on an ultramicro interdigital electrode array chip was used as sensor for anodic stripping voltammetry based detection of heavy metal ions in water [32].

Food- and water-borne pathogens (e.g., viruses, bacteria, and parasites) are the biological agents that cause illness and infectious diseases. The symptoms of the infection vary with their hazardous level, which causes diarrhea to death in extreme cases [33]. Therefore, bacteria detection is a critical process to prevent the infectious disease [34]. Among the various detection techniques, the electrochemical sensing technique is the most used in different sensing applications [35]. Lin et al*.* [36] fabricated an impedimetric biosensor using planar Au- and AuNPs-modified planar Au electrodes followed by self-assembling of thiolated protein G to capture *Escherichia coli* (*E. coli*). Ranjbar et al*.* [37] proposed an electrochemical aptasensor composed of a glassy carbon electrode coated with an AuNPs/carbonNPs/cellulose nanofiber nanocomposite for the detection of *Staphylococcus aureus*. AuNPs-deposited screen-printed electrodes and peptide PEPTIR-1.0 as a recognition molecule for the electrochemical detection of *E. coli* were reported by Ropero-Vega et al*.* [38].

Au nanostructures have been immobilized on different types of substrates and studied the effect of interaction between AuNPs and the substrate, the interparticle spacing upon the sensor sensitivity by optical technique [16, 39]. However, the size and effective surface area of AuNPs on the substrate and the role of interparticle spacing on the current response have not been explored using cyclic voltammetry technique. This study involved three major steps: (1) indium-doped tin oxide (ITO) glass was used as the base substrate, and patterned the electrodes (working electrode (WE), counter electrode (CE), and reference electrode (RE)) was on it; (2) AuNPs were synthesized using two different approaches to achieve two different sizes: trisodium citrate and chitosan-stabilized AuNPs and chitosan-stabilized AuNPs; and (3) synthesized AuNPs were deposited on the working electrode and applied as the detection surface. In this case, two types of gram-negative and gram-positive bacteria, *E. coli* and *E. aurantiacum*, were tested using electrochemical measurement in cyclic voltammetry mode. This study aimed to fabricate a low-cost, portable, and disposable sensor for rapid detection of water-/food-borne pathogens without using specific recognition elements such as enzymes, antibodies, aptamers, and nucleic acids because of the lack of facility at the time being.

## Experimental

### Chemicals and reagents

Sodium chloride (Reagent grade, 99.5%) was obtained from Fluka, Germany. Chitosan (medium molecular weight, 95.6%) was purchased from Sarchem Laboratories, USA. Tetrachloroauric (III) acid trihydrate (HAuCl_4_.3H_2_O, Analytical grade, 99%), potassium ferricyanide and potassium ferrocyanide ((K_3_[Fe(CN)_6_] and K_4_[Fe(CN)_6_]) reagent grade, 99%), and hydrochloric acid (34% w/v, reagent grade) were purchased from Merck, Germany. Trisodium citrate (TSC) (reagent grade, 99.5%) was procured from Fisher Scientific, UK. Nutrient agar was obtained from Difco, USA. Indium tin oxide-coated glass (sheet resistance of ~ 10 Ohms/sq) was procured from Techinstro, India.** S**tandard deionized water (DI) was used throughout the experiment. Sterile distilled water was used for the bacterial culture process.

### Synthesis of gold nanoparticles

#### Synthesis of AuNPs with tri sodium citrate and chitosan

1% (w/v) chitosan solution was prepared using 1% acetic acid (v/v) at room temperature. The prepared chitosan solution (1 mL) was then added to 1 mL of 20 mM HAuCl_4_.3H_2_O. 47.5 mL of DI water was added to the gold-chitosan mixture under constant stirring and heated until boiling. When the solution changed from yellow to pale yellow, 2.5 mL of tri sodium citrate (TSC) solution (56 mM) was added and the mixture continued to boil. After 7 min, the color of the solution turned ruby red, known as the AuNP colloid. The colloidal solution was quenched in an ice bath and stored in a refrigerator for further use. The synthesized sample was denoted as CHI-AuNP-TSC.

#### Synthesis of AuNPs with chitosan

1% chitosan was prepared in 1% acetic acid. The prepared chitosan solution (1 mL) was then added to 1 mL of 20 mM HAuCl_4_.3H_2_O. 45.5 mL of DI water was added to the gold-chitosan mixture, which was boiled under constant stirring. After boiling for 15 min, the color of the solution changed from yellow to ruby red. The solution was quenched in an ice bath and stored in a refrigerator for further use. The synthesized sample was denoted as CHI-AuNP.

### Sensor fabrication process

**Fig. 1:**
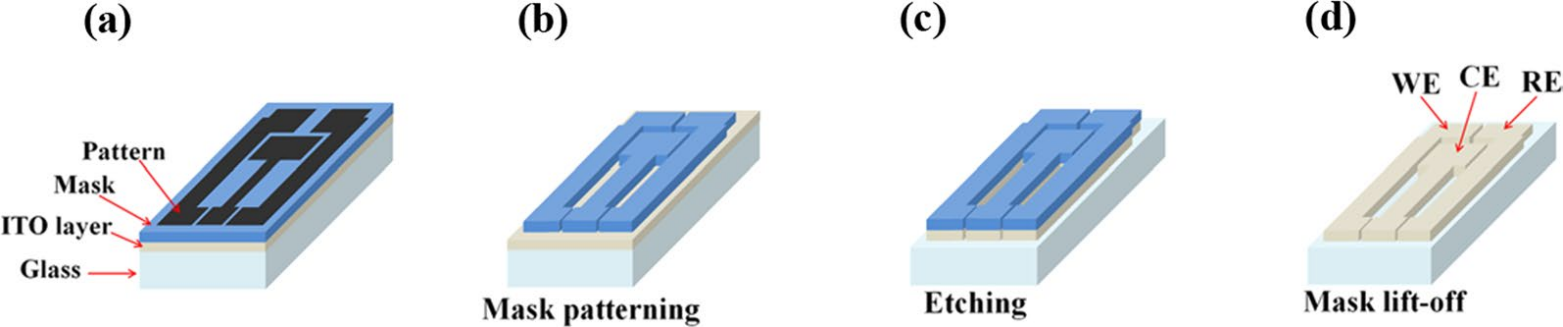
Step-by-step fabrication process of ITO-based three electrodes sensor, **a** Introducing mask design onto ITO substrate which was being masked with vinyl sticker, **b** after patterning the mask, **c** after etching off the unwanted area of ITO surface, and **d** after lifting off the mask to achieve the base of three-electrode sensor’s pattern

**Fig. 2:**
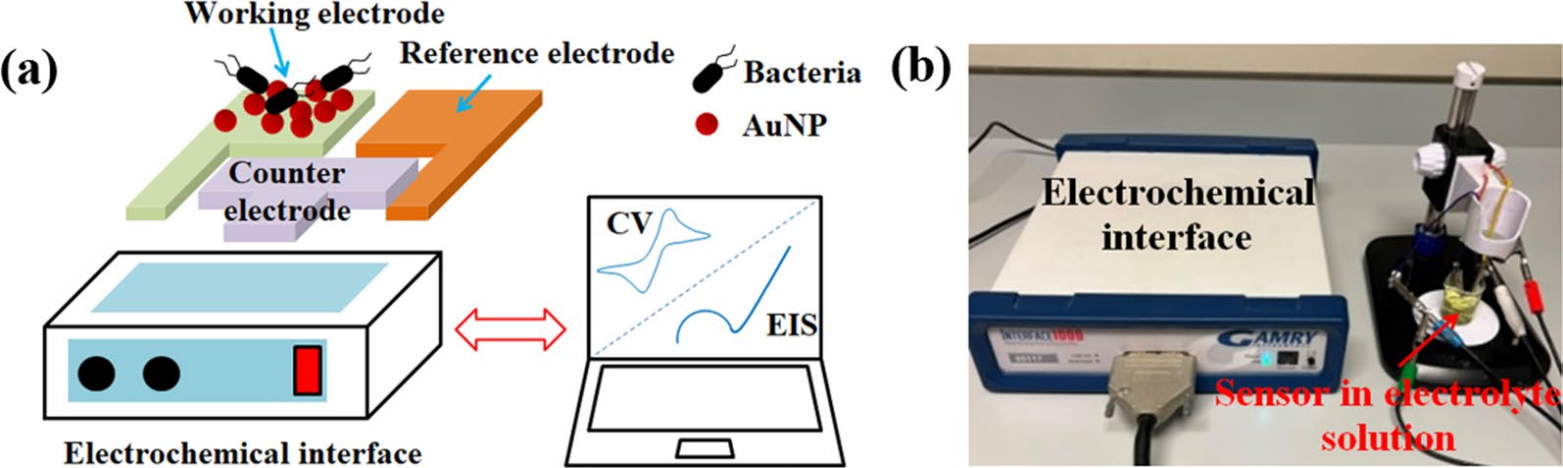
**a** Schematic diagram of three electrodes system sensor test setup and **b** optical image of the electrochemical workstation used in this study for the detection of bacteria using fabricated sensors

**Fig. 3:**
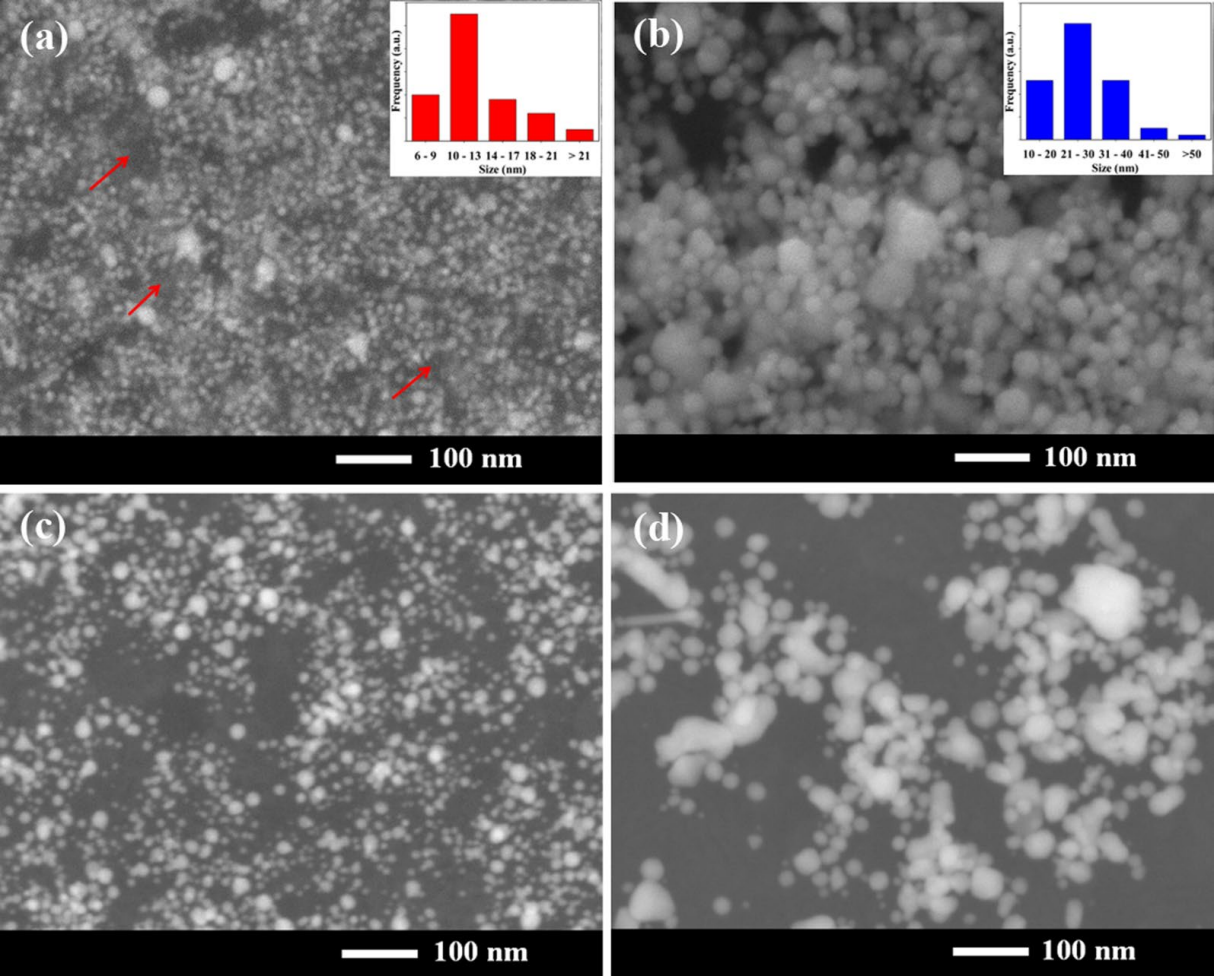
FESEM images of **a** CHI-AuNP-TSC, **b** CHI-AuNP deposited on ITO substrates using secondary electron detector, **c** CHI-AuNP-TSC, and **d** CHI-AuNP using backscattered electron detector. Inset shows the corresponding NPs size distribution histograms of CHI-AuNP-TSC and CHI-AuNP, respectively

Commercially available indium tin oxide (ITO) conducting glass was used as the base substrate, and three sensor electrodes were patterned on it. Four consecutive steps were carried out in this process: (1) design the sensor electrodes according to the position of each electrode (CE, WE, and RE) (see Fig. 1a); (2) transfer the electrode pattern onto masked ITO and thus called mask patterning (see Fig. 1b); (3) etch the unwanted area of the ITO surface to obtain the desired electrode pattern (see Fig. 1c); and (4) lift-off the mask to complete the fabrication process. The sensing areas for the fabricated electrodes were 0.122 cm^2^ for WE and RE but 0.35 cm^2^ for CE which was 2.8 times larger than that of WE and RE, respectively. The WE and RE areas were randomly selected, but a larger electrode area for the CE was intentionally made to deliver the required current for the WE. The apparent sensor dimensions were found to be approximately 13 mm in width and 20 mm in length, with a total area of 2.6 cm^2^ and a thickness of 1 mm. The details of the electrode fabrication processes are provided in supporting information, Fig. S1.

Prior to the sensing performance test, the sensing surfaces were prepared using the following steps. Three microliters of CHI-AuNP-TSC and CHI-AuNP was deposited on the working electrode surfaces, repeated three times, and dried at room temperature. Subsequently, the deposited samples were dried overnight in an oven at 70 °C to enhance the attachment of AuNPs to the working electrode surfaces.

### Preparation of bacteria

First, two types of bacteria, gram-negative bacteria *E. coli* and gram-positive bacteria *E. aurantiacum,* were prepared on the culture plates. The cultured bacteria were transferred to nutrient broth using loops and incubated at 37 °C for 24 h. Serial dilutions were carried out with sterile water before bacterial counting. Subsequently, 0.1 ml of each bacterium was transferred to sterile nutrient agar and kept in an incubator at 37 °C for 24 h. Subsequently, the colony-forming unit per millimeter was calculated (CFU/ml) using the following equation:

CFU/ml = (no. of colonies × dilution factor) / volume of the culture plate.

### Characterizations

Surface morphology of the fabricated EC sensor electrodes was analyzed using a field emission scanning electron microscope (FESEM, JEOL JSM-7800F, Japan). Particle size, morphology, crystal lattice information, and elemental composition were investigated using a high-resolution transmission electron microscopy (HRTEM, JEOL JEM-2100F, Japan). X-ray diffraction was carried out by an X-ray diffractometer (XRD, Rigaku Miniflex-600, Japan), with a step size of 0.02°/s using Cu K_α_ radiation. The optical absorption property of CHI-AuNP-TSC and CHI-AuNP samples was monitored using a UV–visible spectrophotometer (Ocean Optics USB 4000, USA). Attenuated total reflectance Fourier transform infrared spectroscopy (ATR-FTIR) spectrum was conducted by infrared spectroscopy (PerkinElmer SpectraOne, USA). The spectra were acquired in the range of 500–4000 cm^−1^ with a signal resolution of 4 cm^−1^ for 40 scans. The surface and chemical states of the sensor electrodes were studied using X-ray photoelectron spectroscopy (XPS, Scienta Omicron, Germany). Individual components of the obtained XPS spectra were analyzed using a Gaussian Lorentzian with Shirley background function in the Casa XPS software (Casa Software Ltd, UK). The C 1*s* peak with a binding energy value of 246.8 eV was used as a reference for the calibration process. Electrochemical measurements of cyclic voltammetry (CV) and electrochemical impedance spectroscopy (EIS) were conducted using an Interface 1000 potentiostat (Gamry, USA) in the electrolyte solution containing a mixture of 0.5 M NaCl and 1 mM of (K_3_[Fe(CN)_6_]/K_4_[Fe(CN)_6_]) (1:1). The CV tests were performed at a scan rate of 250 mV/s with the scan ranges of − 1 V DC to + 1 V DC. The EIS measurement was carried out in the frequency range from 0.1 Hz to 100 kHz.

### Three electrodes sensor performance evaluation

CHI-AuNP-TSC- and CHI-AuNP-deposited working electrodes were used as sensing components for the detection of bacteria. *E. coli* and *E. aurantiacum* were used as test bacteria. CV was used to study the change in the current response of the sensing surface by varying the concentrations of *E. coli* and *E. aurantiacum* in the electrolyte solution containing a mixture of 0.5 M NaCl and 1 mM of (K_3_[Fe(CN)_6_]/K_4_[Fe(CN)_6_]) (1:1). The CV performance of bacterial sensing was tested at a scan rate of 250 mVs^−1^ with a scan range of − 1 V to + 1 V DC. A schematic diagram of the three-electrode system sensor test setup and the electrochemical workstation is shown in Fig. 2.

## Results and discussion

### Surface morphology of AuNPs on ITO substrate

The morphological features of CHI-AuNP-TSC and CHI-AuNP were determined by FESEM. Figure 3 (a-d) shows the surface morphologies of CHI-AuNP-TSC and CHI-AuNP deposited on ITO substrates, which were taken with two different detectors (secondary and backscattered electron detectors). The isotropic shape AuNPs were mostly observed in both CHI-AuNP-TSC and CHI-AuNP, but a few anisotropic shapes were witnessed in the CHI-AuNP sample. The particle size of CHI-AuNP-TSC ranges from 6 to 25 nm, with the highest particle count for a diameter of 10–13 nm (see Fig. 3a inset). Moreover, AuNPs were well dispersed to form a uniform interparticle spacing on the deposited surface probably due to the presence of the chitosan polymer in its vicinity. (The CHI-dominated area is shown with red arrows.) For the CHI-AuNP sample, the particle size distribution ranges from 10 to 60 nm, with the highest particle count for the diameter of 21–30 nm (see Fig. 3b inset). In this case, AuNPs exhibited an aggregated form whereby the interparticle spacing was not uniform (not well dispersed on the surface). In general, the size of the AuNPs for the CHI-AuNP-TSC sample was much smaller than that of the CHI-AuNP.

**Fig. 4:**
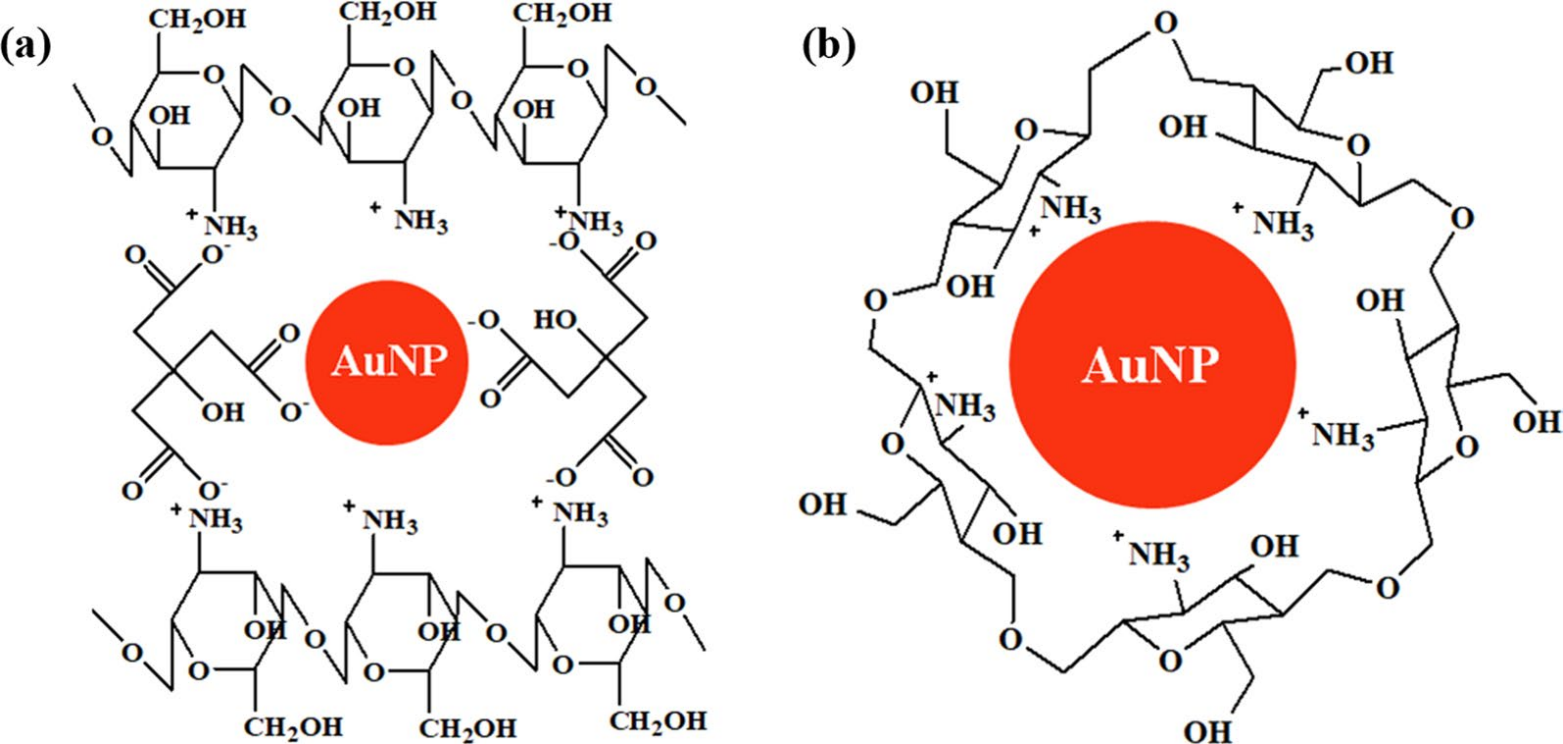
**a** AuNPs surrounded by TSC with cross-linked chitosan and **b** AuNPs surrounded by chitosan

From the aforementioned observations, it can be suggested that TSC in the AuNPs synthesis process affects two scenarios: size (direct) and particle spacing (indirect). First, the smaller size of NPs in CHI-AuNP-TSC was due to the strong reducing behavior of TSC (main reducing agent), which could favor the faster reduction of AuCl_4_^−^ to Au^0^ [40]. Second, chitosan performed as a capping agent for AuNPs during the synthesis process resulting in smaller sizes of NPs, and the excessive chitosan (a portion of chitosan might serve in the reducing process) served as a matrix to attract AuNPs by electrostatic interaction [41] (as illustrated in Fig. 4a). This chitosan matrix also acted as a film to achieve a well-dispersed AuNPs layer on the deposited surface (see Fig. 3a). However, a larger size of AuNPs was witnessed in the CHI-AuNP sample as chitosan served as both a reducing and stabilizing/capping agent at the same time (see Fig. 4b), and the formation of gold nanoparticles took a longer time than the CHI-AuNP-TSC (Two times higher). In this case, most of the chitosan was consumed during the synthesis process, and less chitosan served as a capping agent. Thus, when CHI-AuNP-TSC and CHI-AuNP were deposited on the ITO substrate, the interparticle spacing of the CHI-AuNP-TSC sample was well organized and uniform compared to that of the CHI-AuNP sample. This could be due to the film-forming ability of CHI in CHI-AuNP-TSC whereby it prevents the aggregation of AuNPs on the ITO substrate (see Fig. 3a). On the other hand, most of the chitosan was consumed as both a reducing and capping agent and no additional chitosan was left to form a film in the CHI-AuNP sample. Therefore, less interparticle spacing with aggregation of AuNPs was witnessed on the ITO substrate (see Fig. 3b and d).

### The morphology, particle size, and elemental composition of AuNPs

**Fig. 5:**
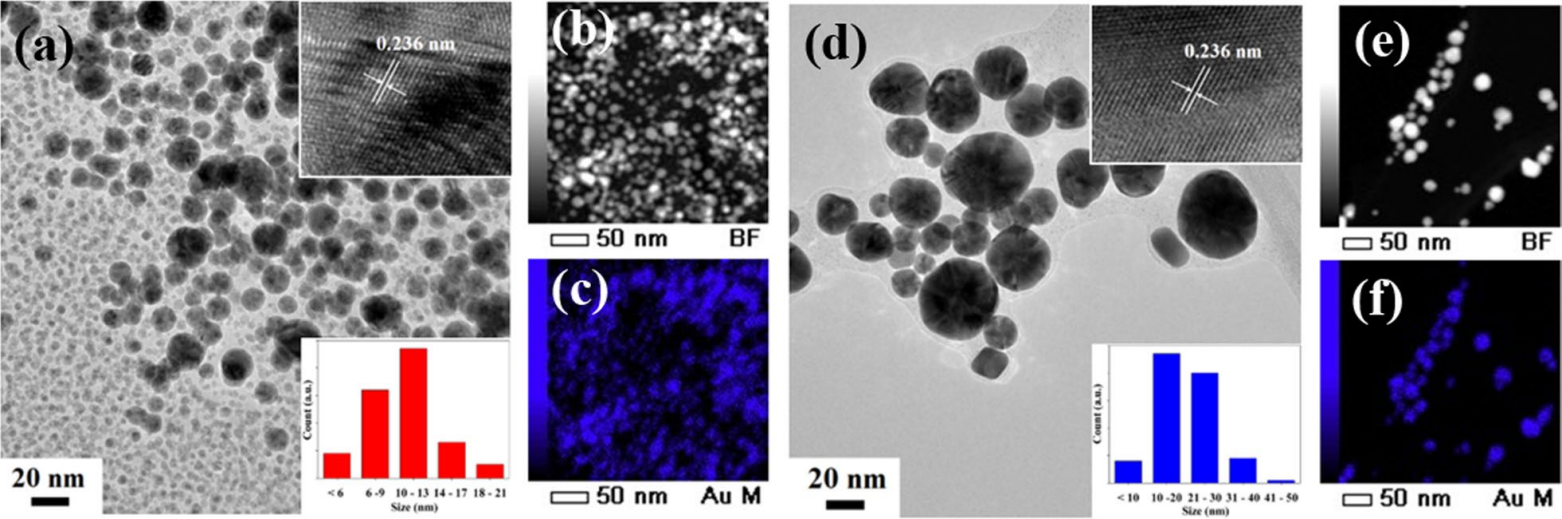
HRTEM images of **a** CHI-AuNP-TSC (inset: lattice spacing and size distribution histogram), **b** bright-field image, **c** EDS mapping, **d** CHI-AuNP (inset: lattice spacing and size distribution histogram), **e** bright-field image, and **f** EDS mapping

The morphology, crystal structure, size, and composition of the synthesized AuNPs were further investigated using HRTEM. Figure 5 shows the TEM image of CHI-AuNP-TSC and CHI-AuNP samples. CHI-AuNP-TSC sample exhibited isotropic shape nanoparticles with two distinct-size groups (particle size < 6 nm; particle size between 7 and 20 nm), and narrow size distribution was observed. For CHI-AuNP, one type of size group with a bigger particle size was noted compared with CHI-AuNP-TSC. Lattice spacing for both of the samples (inset: Fig. 5a and d) was found to be ~ 0.236 nm which was from (111) interplanar distance of FCC Au crystal [42]. The particle size ranges from 3 to 20 nm with the highest particle count of 10–13 nm for CHI-AuNP-TSC, and 7 nm to 40 nm with the highest particle count of 10–30 nm was observed for CHI-AuNP (Fig. 5a–d inset: particle size distribution graph). In contrast, particle size distribution estimated from the TEM result was slightly different (smaller size in TEM than SEM) from SEM (Fig. 3a–b inset). This could be due to the difference in the resolution (0.8 nm for SEM and 0.1 nm for TEM), imaging technique (secondary electron detection for SEM and transmission electron imaging for TEM), and sample preparation method [43]. However, EDS mapping showed the expected materials like Au (see Fig. 5b, c, e, and f), C, and O from the polymer material and TEM grid (C and O data are not shown here).

### Crystalline and surface functional group of synthesized AuNPs

**Fig. 6:**
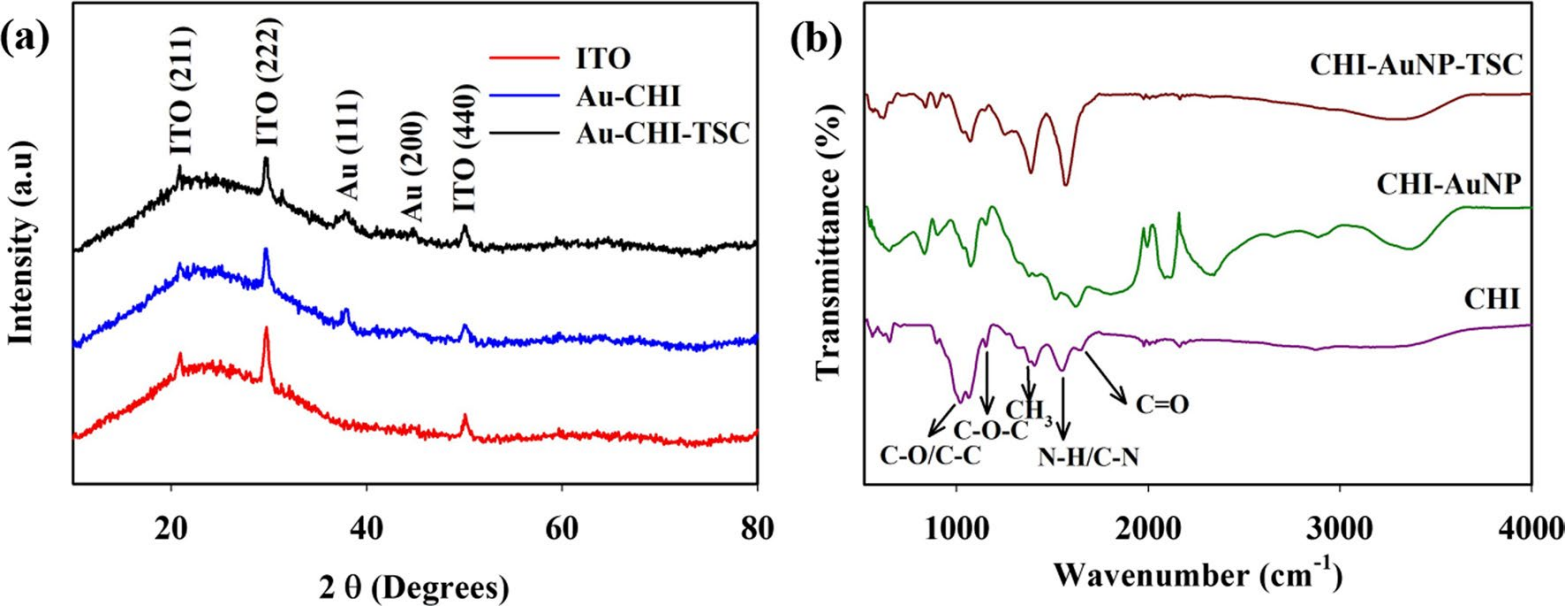
**a** The XRD patterns of bare ITO, CHI-AuNP-TSC, and CHI-AuNP deposited on ITO substrates and **b** FTIR spectra of as synthesized CHI-AuNP, CHI-AuNP-TSC, and pristine CHI deposited on ITO substrates

The crystalline structure of AuNPs was analyzed using XRD. Figure 6 displays the XRD patterns of CHI-AuNP-TSC and CHI-AuNP deposited on the ITO substrates. Note that the ITO substrate was included as a reference for the comparison. The XRD patterns of ITO showed the characteristic peaks of ITO located at 2*θ* = 21.0°, 29.9°, and 50.0° for the indexing angles of reference planes at (211), (222), and (440), respectively [44]. Moreover, two additional peaks at 38.1° and 44.3° were observed in CHI-AuNP-TSC and CHI-AuNP, respectively, which can be assigned to Au (111) and (200) [45]. These observed planes affirmed that AuNPs were deposited on ITO substrates. Besides, Au (111) peak was broadening in CHI-AuNP-TSC, which indicated that AuNPs were smaller in CHI-AuNP-TSC than in CHI-AuNP [46]. These findings were consistent with the FESEM and HRTEM results (see Figs. 3 and 5). The estimated crystalline size of CHI-AuNP-TSC and CHI-AuNP was found to be 0.71 nm and 2.84 nm, respectively.

FTIR spectroscopy was employed to study the functional groups of CHI-AuNP-TSC and CHI-AuNP samples, and pristine CHI was used as a reference/comparison (see Fig. 6b). The omnipresence of chitosan was noted by the exhibition of similar peaks in both the CHI-AuNP-TSC and CHI-AuNP samples when compared with the pristine CHI sample. The stretching vibrations of O–H and N–H groups were observed at around 3380–3340 cm^−1^, while the absorption band at 2880–2870 cm^−1^ reflected the symmetric and asymmetric stretching of C–H bonds. The presence of C=O in the amide I group, CH_3_ in the amide, and C–OH group was also confirmed by absorption bands at 1650–1550 cm^−1^, 1370–1390 cm^−1^, and 1250–1260 cm^−1^, respectively. Furthermore, two bands appearing at 1150–1160 cm^−1^ and 1552 cm^−1^ were the bending of N–H bonds and C–N stretching, which affirmed the presence of the ether group of chitosan. C–O stretching of primary alcohol was observed at 1072 cm^−1^ for CHI-AuNP-TSC and CHI-AuNP samples, while it was also found in pristine CHI sample by detecting the dominant C–O stretching broad bands at 1066 cm^−1^ and 1018 cm^−1^, respectively [47–50]. The presence of AuNPs in the CHI matrix caused interactions by observing the peaks shifting or changing. For instance, the absorption bands of O–H/N–H and C=O of the CHI-AuNP and CHI-AuNP-TSC samples shifted when compared with the pristine CHI (Fig. 6b). Moreover, the formation of the positive absorption frequency around 2000 cm^−1^ was witnessed for the CHI-AuNP sample, probably owing to the interference of IR excitation with the plasmon resonance frequency of larger AuNPs. The existence of C–H, N–H, C=O, and C–O was also further confirmed by XPS analysis as described in section "Surface properties of AuNPs on ITO substrate."

### The optical properties of AuNPs on ITO substrate

**Fig. 7:**
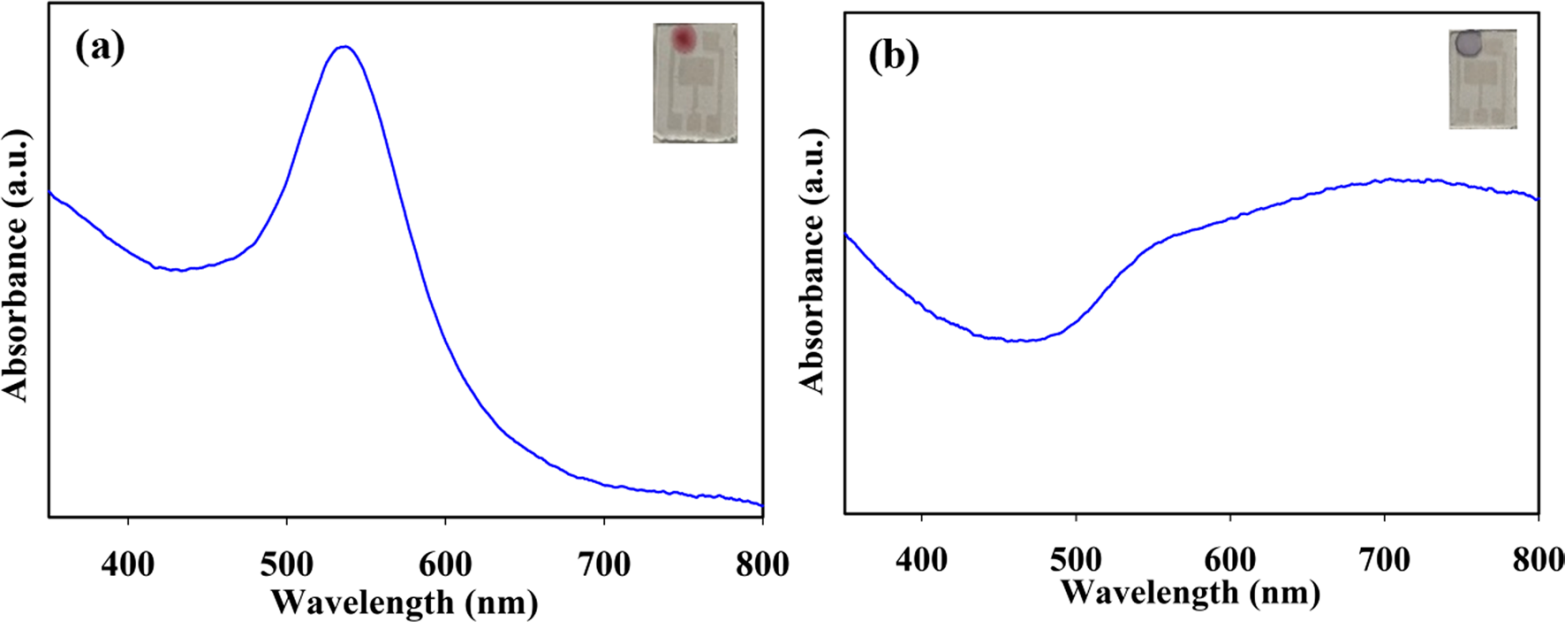
UV–visible absorption spectra of **a** CHI-AuNP-TSC and **b** CHI-AuNP deposited on ITO substrates (insets show the optical image of substrate (ITO)-bound AuNPs)

The optical properties of AuNPs were studied using UV–visible spectroscopy. Figure 7a and b illustrates the absorption spectra of CHI-AuNP-TSC and CHI-AuNP deposited on ITO substrates. The maximum absorption peak of the CHI-AuNP-TSC (Fig. 7a) emerged at 537 nm due to the localized surface plasmon resonance of AuNPs. The inset in Fig. 7a shows an optical image of AuNPs-deposited ITO sensor substrate and can be visually observed as a red color. The CHI-AuNP in Fig. 7b shows an absorption spectrum with a plateau that starts at a wavelength of 500 nm and continues to a higher wavelength. The blue color of the sample can be seen visually for the CHI-AuNP sample deposited on the ITO sensor substrate (Fig. 7b inset). The difference in the color of the samples indicated a difference in the size and formation of the AuNPs on the ITO substrates. The red color of the CHI-AuNP-TSC sample shows a smaller size of AuNPs with a certain interparticle spacing, while the blue color of the CHI-AuNP sample indicates a larger size of AuNPs with a very small interparticle spacing on ITO substrates (see Fig. 3a and b). The dipole–dipole interactions between AuNPs could lead to a coupling effect in which a larger interparticle distance is associated with a weaker coupling effect, and a narrower SPR absorption band [20, 51]. The UV–visible absorption spectra were consistent with the SEM images, where certain interparticle spaces were witnessed in the CHI-AuNP-TSC (see Fig. 3a). In the CHI-AuNP sample, the NPs were larger and the interparticle distances were also very small due to the aggregated structure of AuNPs on the ITO surface (see Fig. 3b). The experimental results were in good agreement with the reported studies of noble NPs in various host matrices, according to which the position of SPR peak changes upon changing of the surrounding environment [52].

### Surface properties of AuNPs on ITO substrate

**Fig. 8:**
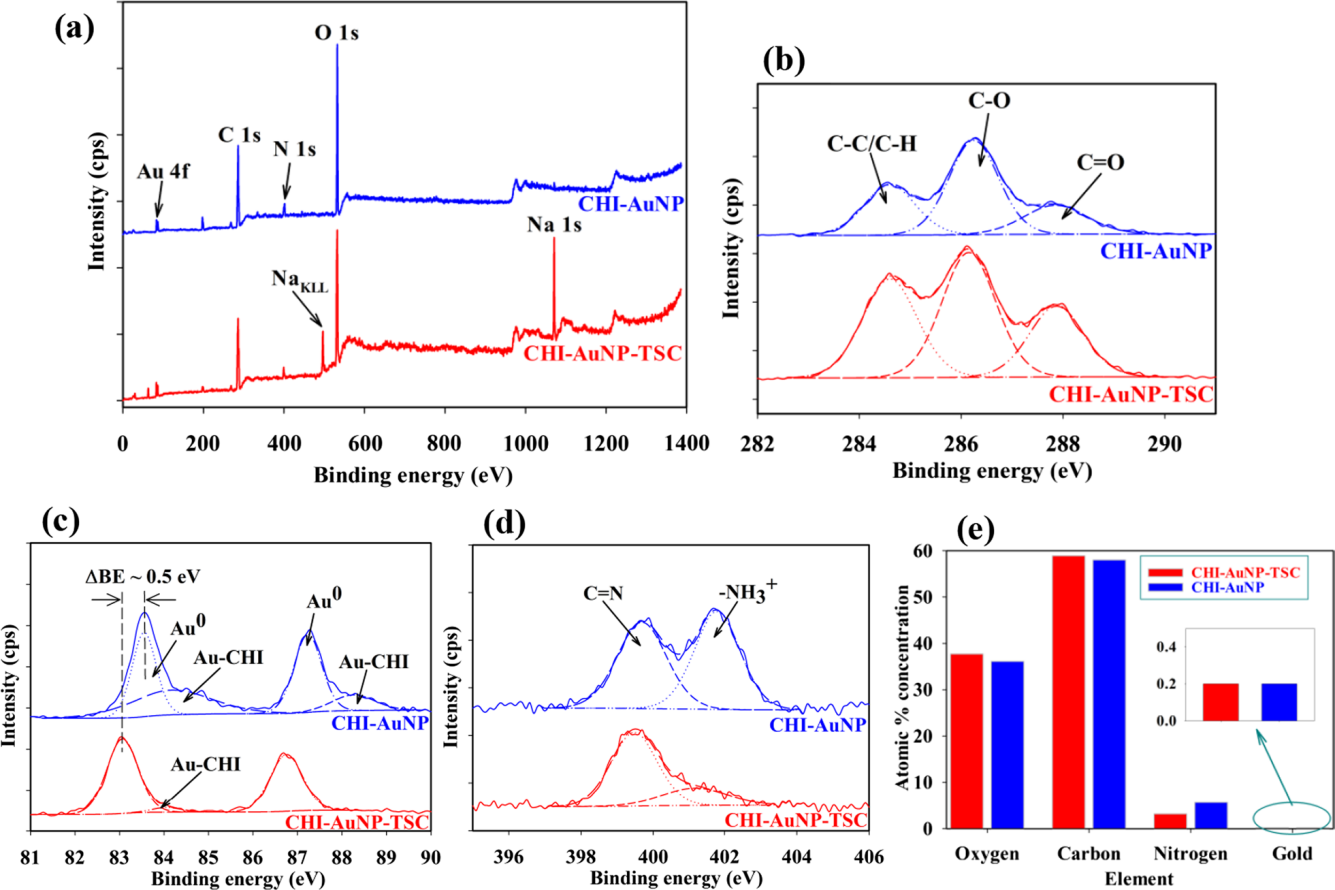
**a** XPS survey spectrum, **b** core-level high-resolution C 1*s* peaks, **c** core-level high-resolution Au 4f peaks, **d** core-level high-resolution N 1*s* peaks, and **e** quantitative analysis of O, C, N, and Au for CHI-AuNP-TSC and CHI-AuNP samples

Figure 8a displays the surface and sub-surface survey spectra for the CHI-AuNP-TSC and CHI-AuNP deposited on ITO substrates. Core-level Au 4f, C 1*s*, O 1*s*, and N 1*s* peaks were detected on both of the samples, but extra peaks of Na 1*s* and Na_KLL_ (Auger) peaks were observed on TSC reduced AuNPs surface. The presence of a high concentration of C 1*s* and N 1*s* peaks revealed the existence of a chitosan matrix in the vicinity of AuNPs, which was further confirmed by the BE shift in core-level Au 4f peaks. Core-level C 1*s* peaks for both CHI-AuNP-TSC and CHI-AuNP samples (Fig. 8b) composed with C–C/C–H where BE centered at 284.6 eV, C–O at 286.2 eV, and C=O at 287.8 eV, respectively [53]. Figure 8c shows the core-level Au 4f peaks with spin–orbit splitting for Au 4f_7/2_ at 83.0 eV and Au 4f_5/3_ at 86.7 eV, respectively. Two obvious differences were witnessed between CHI-AuNP-TSC and CHI-AuNP samples. First, two distinct Au 4f peaks with shake-up satellite peaks positioned at 84.2 eV and 87.2 eV for CHI-AuNP sample which displayed the coexistence of Au^0^ and Au-CHI (CHI terminated AuNPs) whereby CHI-AuNP-TSC showed very small extra peak at BE value of 84 eV. The enhanced Au-CHI peaks in CHI-AuNP were due to the strong interaction between AuNPs and CHI, where CHI served as both reducing and capping agent, as more CHI interacted with AuNPs in CHI-AuNP sample (see Fig. 4b). Second, the core-level Au 4f peak shifted to higher BE for the CHI-AuNP sample which could be due to the complete electron transfer from AuNPs to the surrounding chitosan matrix [54]. This result affirmed the interaction between AuNPs and chitosan for CHI-AuNP-TSC and CHI-AuNP (see Fig. 4). For instance, AuNPs were surrounded by a chitosan matrix with weakly bound for CHI-AuNP-TSC (see Fig. 4a), but there were complete electrons or charges transferred for CHI-AuNP (see Fig. 4b).

For core-level N 1*s* peaks (Fig. 8d), two distinct peaks with BE values of 401.8 eV for C=N and 399.7 eV for –NH_3_^+^ were observed for CHI-AuNP but very low peak intensity for –NH_3_^+^ was detected for CHI-AuNP-TSC. These substantial increases in –NH_3_^+^ indicated the presence of chitosan at a very close proximity to AuNPs, especially for aggregated fashion in the CHI-AuNP sample (see Figs. 3b and d and 4b). Conversely, CHI-AuNP-TSC has less nitrogen content due to the presence of TSC on the AuNPs' surface. This phenomenon was confirmed by witnessing the higher content of C and O in the CHI-AuNP-TSC sample (Fig. 8e). The concentration of AuNPs (in terms of atomic % concentration) for both samples was the same, which indicated the total amount of Au present in the sensor surface could be the same for both samples. These above findings shed light that the amount of Au present on the surface did not play a role in improving the bacteria sensing property, but the size and distribution of AuNPs on the surface did the enhancement of bacteria detection, which will be discussed in section "Bacteria detection by electrochemical approach." However, more studies need to be done to prove the aforementioned above claim.

### Electrochemical performance of AuNPs on ITO substrate

**Fig. 9:**
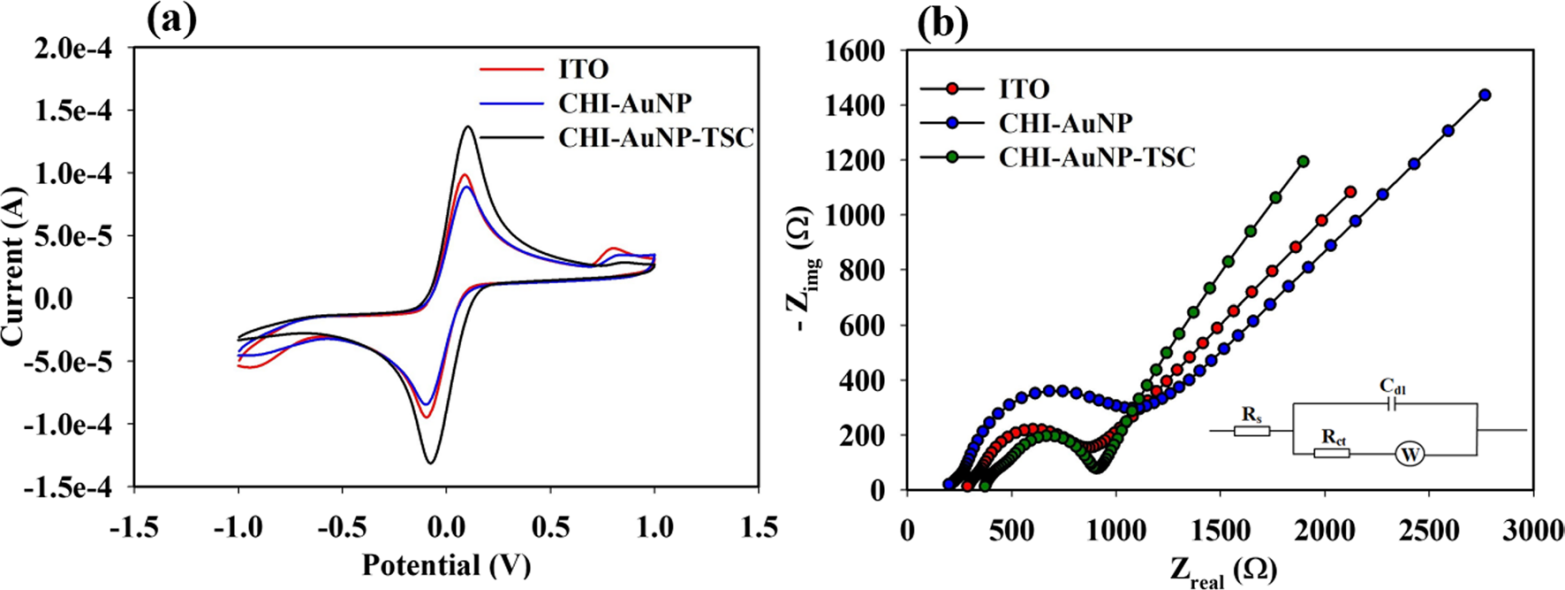
**a** Cyclic voltammograms of bare ITO, CHI-AuNP-TSC on ITO, and CHI-AuNP on ITO and **b** Nyquist plot of bare ITO, CHI-AuNP-TSC on ITO, and CHI-AuNP on ITO; inset shows the equivalent circuit for fitting the Nyquist of all electrodes

Voltammetry sweep curves were obtained using bare ITO, CHI-AuNP-TSC and CHI-AuNP deposited on the ITO working electrodes (also called the sensing electrodes). The electrochemical behavior was tested in a mixture of 0.5 M NaCl and 1 mM K_3_[Fe(CN)_6_]/K_4_[Fe(CN)_6_] electrolyte solution. The CV curve of ITO was used as a reference for a comparison with those of the CHI-AuNP-TSC and CHI-AuNP sensing electrodes. According to the cyclic voltammograms in Fig. 9a, a characteristic pair of redox peaks was obtained for the bare ITO with a peak potential separation (ΔE_p_ = E_pa_ − E_pc_) of 180 mV, corresponding to the well-known quasi-reversible one-electron transfer redox behavior of the K_3_[Fe(CN)_6_]/K_4_[Fe(CN)_6_] redox couple. The CHI-AuNP-modified ITO electrode also exhibited the same redox map but with a wider (ΔE_p_ = 195 mV) and lower current response than the bare ITO. For CHI-AuNP-TSC-modified ITO electrode, an improved current response with wider peak potential separation (ΔE_p_ = 225 mV) was obtained. Moreover, a small additional oxidation peak at ~ 0.8 V and a reduction peak at ~ 0.9 V (vs Ag/AgCl) could be due to the ITO substrate–redox probe interaction. After the deposition of CHI-AuNP and CHI-AuNP-TSC on the ITO surface, reduction of these additional peaks was observed because of less interaction between the ITO surface and redox probe. The improvement in the current response of the CHI-AuNP-TSC was an indication of the significant enhancement of the electron transfer kinetics, which could be due to the higher active surface area of AuNPs in the CHI-AuNP-TSC sample. The smaller size of AuNPs with larger interparticle spacing in the CHI-AuNP-TSC sample (see Fig. 3a and c) orchestrated the higher surface area (~ 6.26 × 10^–13^ m^2^ per specific area, which is 30% higher than the original surface area, see supporting information) than those of the smaller interparticle spacing with larger NPs sizes in the CHI-AuNP sample (~ 5.57 × 10^–13^ m^2^ per specific area, which is 16% higher than the original surface area, see supporting information). This circumstance was one of the key factors to enhance the sensing of bacteria as will be discussed in the further section.

Electrochemical impedance spectroscopy (EIS) is an electrochemical tool for analyzing the interfacial charge transfer properties of the CHI-AuNP-TSC and CHI-AuNP electrodes. In this work, the impedimetric behavior of the prepared electrodes was investigated using a mixture of 0.5 M NaCl as the electrolyte and 1 mM K_3_[Fe(CN)_6_]/K_4_[Fe(CN)_6_] as a redox probe. The Nyquist plots in Fig. 9b comprise two components: semicircles in the high-frequency region and a linear part in the low-frequency region. The semicircle in the high-frequency attributed to the electron transfer process, while the linear part in the lower-frequency ascribed to transport limitations of electroactive species diffuse to the reaction interface known as Warburg impedance [41]. The diameter of the semicircle represents the charge transfer resistance (*R*_ct_) that usually forms at the electrode/electrolyte interface [55]. From the Nyquist plot shown in Fig. 9b, the *R*_ct_ value of CHI-AuNP-TSC (408 Ω) was lower than that of bare ITO (480 Ω) and AuNP-CHI (724 Ω). This lower *R*_ct_ could be due to the higher surface area (smaller nanoparticle size) as well as the existence of interparticle spacing between AuNPs in the CHI-AuNP-TSC sample (see Fig. 3a and c), which improved the surface conductivity and heterogeneous electron transfer kinetics activity. This phenomenon was consistent with the CV data shown in Fig. 9a, where the CHI-AuNP-TSC sample achieved the maximum current. In contrast, the series resistance (R_s_) of CHI-AuNP (232 Ω) was smaller than that of CHI-AuNP-TSC (401 Ω) and bare ITO (314 Ω). Generally, R_s_ is the resistance of the electrolyte along with the internal resistance of the electrode (bare ITO electrode and AuNPs deposition layer). In our case, the electrolyte resistance and the current collector were considered to be uniform, but the conductivity of the ITO could be altered according to the area of the working electrodes. Even though all the working electrodes kept the same area, a slight variation occurred during manual masking (mentioned in the electrode preparation section); thus, different *R*s values were obtained for the bare ITO electrodes (see Fig. S2, supporting information). However, the remarkable decrease in *R*_ct_ for the CHI-AuNP-TSC greatly influenced the sensing performance, which will be discussed in the next section. The Nyquist plots were fitted with the equivalent circuit as shown in Fig. 9b, inset.

### Bacteria detection by electrochemical approach

**Fig. 10:**
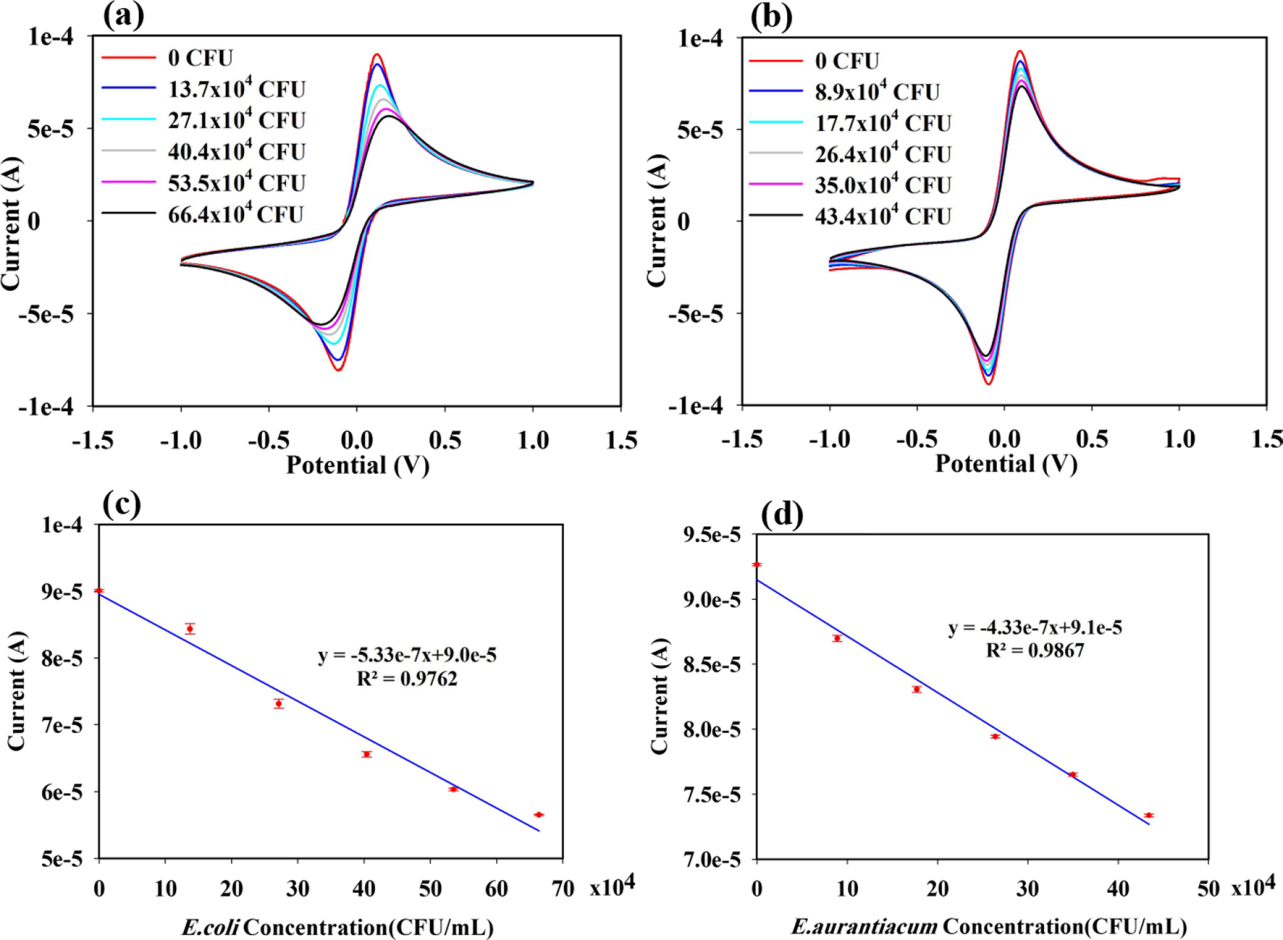
CV performance of CHI-AuNP-TSC sensing electrode at different concentrations of **a** *E. coli*, **b** *E. aurantiacum*, **c** the linear slope of the anodic peak current vs. *E. coli* concentrations, and **d** the linear slope of the anodic peak current vs. *E. aurantiacum* concentrations

To determine the sensing performance of CHI-AuNP-TSC- and CHI-AuNP-deposited working electrodes, two commonly known gram-negative and gram-positive bacteria: *E. coli* and *E. aurantiacum* were used. Figure 10a and b shows the CV performance of CHI-AuNP-TSC electrode at different concentrations of *E. coli* from 14 × 10^4^ CFU/mL to 66 × 10^4^ CFU/mL and *E. aurantiacum* from 9 × 10^4^ CFU/mL to 44 × 10^4^ CFU/mL, respectively. Before adding the bacteria, the highest anodic and cathodic peak currents were ~ 90 μA for CHI-AuNP-TSC and CHI-AuNP sensors. Although both anodic and cathodic peak currents formed during the oxidation and reduction process, the anodic peak current was mainly used to determine the sensing performance. After adding 50 μl of *E. coli* (to make 14 × 10^4^ CFU/mL) and *E. aurantiacum* (to make 9 × 10^4^ CFU/mL) to the electrolyte solution, the values of the anodic peak current (Ipa) reduced from 90 μA to 85 μA for *E. coli* and 87 μA for *E. aurantiacum*. When both bacteria concentrations were further increased by adding 250 μl to the electrolyte solution, the current response of both sensing surfaces achieved 57 μA for *E. coli* and 74 μA for *E. aurantiacum*, which were reduced by 37% and 20% from that of initial Ipa values. The plot of anodic peak current associated with different concentrations of bacteria exhibited a linear trend as shown in Fig. 10c and d. Moreover, the peak potential separation was more obvious in *E. coli* (ΔE_p_ ~ 394 mV) than in *E. aurantiacum*, especially in a higher concentration of bacteria. Therefore, it can be concluded that the CHI-AuNP-TSC sensing surface was more selective toward *E. coli* than *E. aurantiacum*. In order to confirm the current response changes due to the detection of bacteria, different volumes of nutrient broth (50 μl to 250 μl) without bacteria were added to the electrolyte solution (Fig. S3). It was clearly seen that no obvious reduction in current response was observed for both CHI-AuNP-CTS and CHI-AuNP samples, confirming that the reduction in current response was triggered mainly due to the presence of bacteria.

**Fig. 11:**
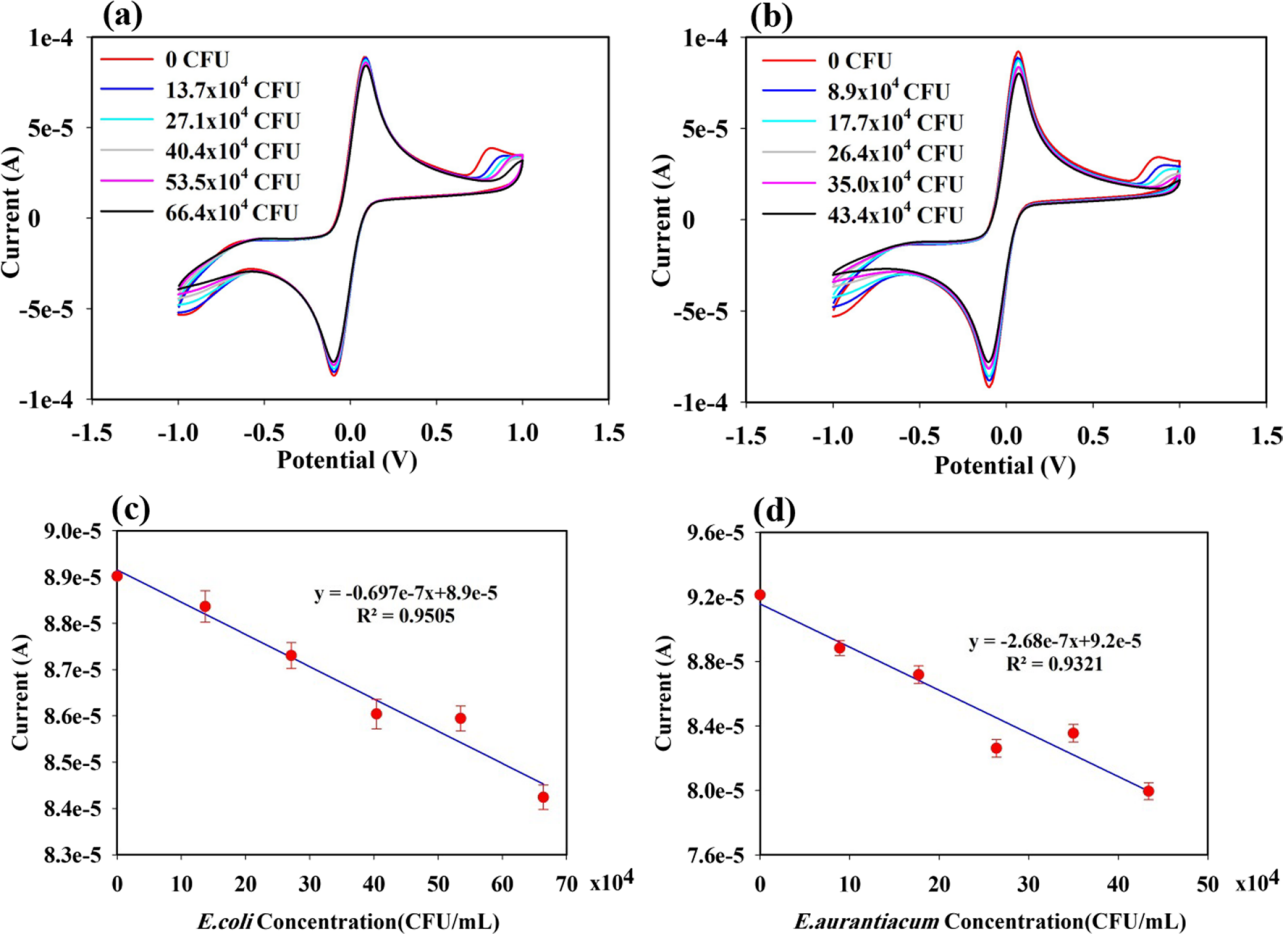
CV performances of CHI-AuNP sensing electrode at different concentrations of **a** *E. coli*, **b** *E. aurantiacum*, **c** the linear slope of the anodic peak current versus *E. coli* concentration, and **d** the linear slope of the anodic peak current versus *E. aurantiacum* concentration

Figure 11a and b shows the CV graphs of sensing performance which were conducted in the same way as discussed above using CHI-AuNP electrodes. After introducing different concentrations of *E. coli* and *E. aurantiacum*, the reduction of current response was observed in both cases (see Fig. 11a and b). The current response for *E. coli* decreased from 89 μA (no bacteria) to 84 μA (66 × 10^4^ CFU/mL) and for *E. aurantiacum* decreased from 92 μA (no bacteria) to 80 μA (44 × 10^4^ CFU/mL), respectively. The anodic peak current decreased by 5.6% for *E. coli* and 13% for *E. aurantiacum* from that of the initial Ipa values. CHI-AuNP sensing surface was less selective for *E. coli* than *E. aurantiacum* when compared with the CHI-AuNP-TSC sensing performance. Figure 11c and d shows the linear relationship of a slope of the anodic peak current related to the different concentrations of bacteria. The limit of detection (LOD) for CHI-AuNP-TSC and CHI-AuNP was estimated using LOD = 3S/M where S represents the standard deviation of the Ipa and M stands for the slope of the Ipa vs. different concentrations of both bacteria [56]. The LOD for *E. coli* and *E. aurantiacum* of the CHI-AuNP-TSC sensor was 1.07 CFU/mL and 2.41 CFU/mL, and the CHI-AuNP sensor was 12.68 CFU/mL and 5.22 CFU/mL, respectively. In addition, a comparison of the detection limits between this work and the reported studies using electrochemical techniques is presented in Table 1.

**Table 1:** A comparison of the detection limits between this work and the reported studies

Type of Pathogen	Biorecognition element	Type of electrode	LOD (CFU/mL)	Reference
*E. coli* *and E. aurantiacum*	–	CHI- and TSC-capped AuNPs on ITO	1.1 and 2.4	This work
*E. coli* *and E. aurantiacum*	–	CHI-capped AuNPs on ITO	12.7 and 5.2	This work
*Staphylococcus aureus*	Aptamer	AuNPs/carbon NPs/cellulose nanofibers GC	1.0	[37]
*E. coli* O157:H7	–	ZnO nanorods on carbon ink-printed glass substrate	1.0 × 10^3^	[57]
*Salmonella typhimurium*	Anti-*S*. *typhimurium* HRP	CHI on GC	35.0	[58]
*Aspergillus niger*	–	ITO	2.0 × 10^3^	[59]
*Salmonella typhimurium*	Poly-dopamine	Gold screen-printed electrode	47.0	[60]
*Salmonella*	Anti-*Salmonella*	CoFe–MOFs–graphene on GC	120.0	[61]
*Salmonella pullorum and Salmonella gallinarum*	Anti-*S. pullorum* and anti-*S. gallinarum*	AuNPs on screen-printed electrode	3.0 × 10^3^	[62]

From the sensing performances of CHI-AuNP-TSC and CHI-AuNP shown in Fig. 10 and Fig. 11, the reduction of peak current response after adding the bacteria was due to the charge transfer inhibiting molecules (bacteria) toward the vicinity of the sensing electrode [13]. In other words, when the K_3_[Fe(CN)_6_]/K_4_[Fe(CN)_6_] was oxidized/reduced at the surface of the electrode, the redox species along with bacteria were moving toward the sensing surface. The flux of the redox species was disturbed by the presence of bacteria near or on the sensing electrode surface resulting in the reduction of the peak current was obtained. The presence of bacteria on the electrode surface was further confirmed by SEM which will be discussed in detail in a later section. However, the current response in CHI-AuNP-TSC (Fig. 10) was higher than CHI-AuNP (Fig. 11) for both types of bacteria. This could be due to the combined effect of CHI and AuNPs in the CHI-AuNP-TSC sample where smaller AuNPs with interparticle spacing could lead to a higher electroactive surface area (see Fig. 3a and c). This phenomenon was confirmed by observing the improvement in surface conductivity and good electron transfer ability (confirmed by the small *R*_ct_ value in Fig. 9b) of the CHI-AuNP-TSC sensing surface and thus a higher redox current response. In addition, both bacteria have negative charge although they have different nature of outer cell membrane. Gram-negative bacteria (*E. coli*) are composed of a thin layer of lipopolysaccharide at the outer surface, followed by another peptidoglycan layer of ~ 7 to 8 nm. The cell walls of gram-negative bacteria lack strength and rigidity which can provide more anchoring sites to promote more negative charges on them. In contrast, the cell walls of gram-positive bacteria (*E. aurantiacum*) are rigid and comprise a thick outer membrane peptidoglycan layer of ~ 20 to 80 nm. The thicker membrane provides fewer anchoring sites resulting in fewer negative charges on gram-positive bacteria [63, 64]. The AuNPs-embedded-CHI matrix on the sensing surface in CHI-AuNP-TSC sample (see Figs. 3a and 4a) might attract bacteria through electrostatic attraction, thus enhancing the bacterial sensing activity, especially for gram-negative bacteria (*E. coli*). In contrast, the aggregated structure with larger AuNPs promotes less electroactive surface area in the CHI-AuNP sample, which led to lower redox current response and sensitivity toward both types of bacteria.

### Post-analysis of the sensing surfaces

**Fig. 12:**
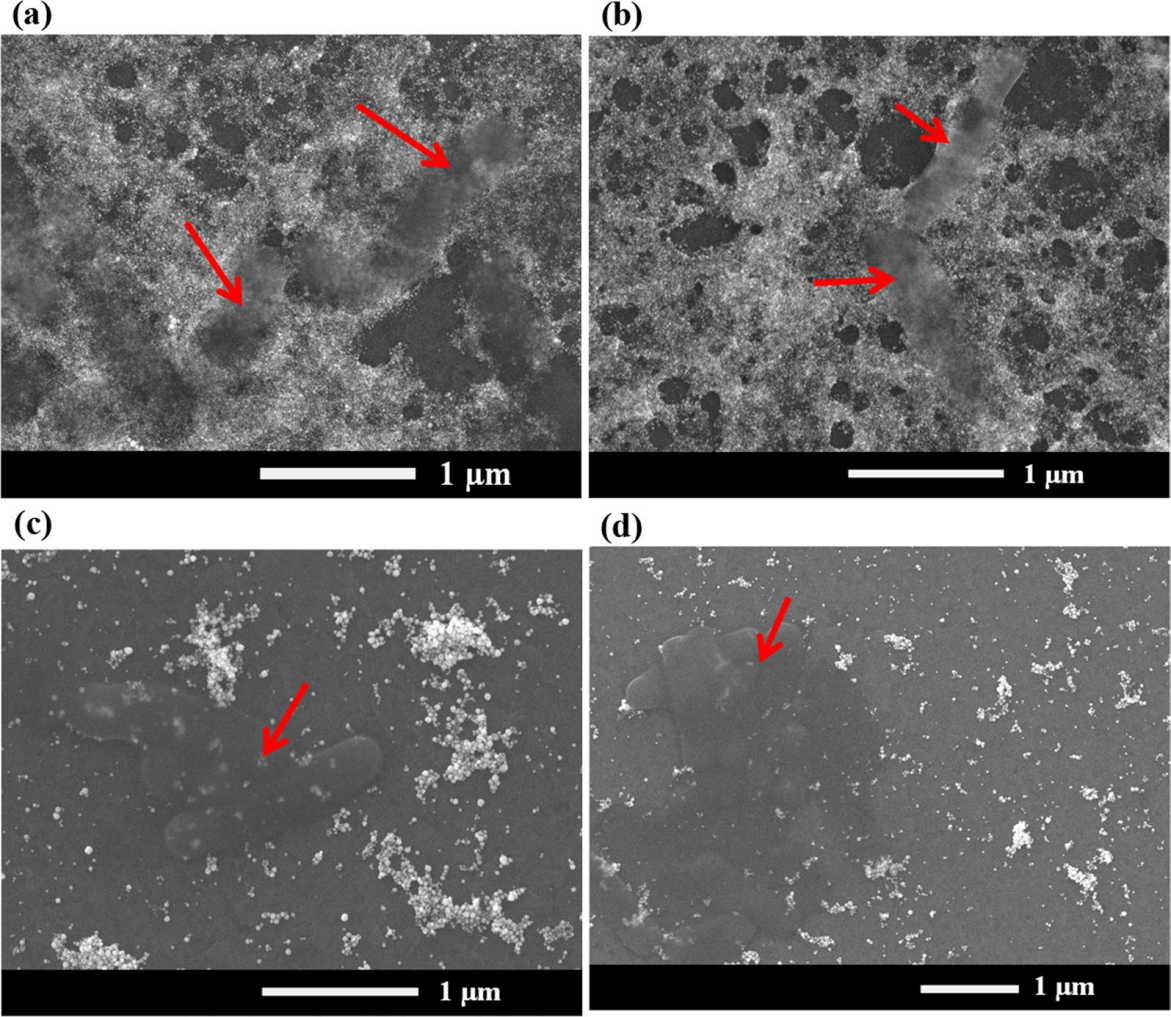
FESEM images of the presence of bacteria on sensing surfaces **a** *E. coli*, **b** *E. aurantiacum* on CHI-AuNP-TSC, **c** *E. coli*, and **d** *E. aurantiacum* on CHI-AuNP

Post-analysis of used CHI-AuNP-TSC and CHI-TSC sensing surfaces was conducted by using FESEM. Prior to the analysis, the used sensing surfaces were gently cleaned with DI water to remove the deposited salt from the electrolyte, and dried at room temperature. Figure 12a-d clearly exhibits the presence of bacteria (both *E. coli* and *E. aurantiacum*) on both of the CHI-AuNP-TSC and CHI-TSC sensing surfaces (shown by red arrows). This observation of bacteria confirmed our previous statement of the movement of bacteria along with the redox species toward the sensing surfaces during oxidizing/reducing reaction. However, the number of bacteria on the CHI-AuNP-TSC and CHI-AuNP sensing surfaces do not reflect the redox current response (Figs. 10 and 11). Moreover, bacteria were still intact (without disintegrating) on both sensing surfaces, suggesting that our sensor's detection phenomenon was non-destructive. After utilizing the sensor for bacteria detection, a noticeable amount of AuNPs was still present on both sensing surfaces. Nevertheless, fewer amounts of AuNPs were observed on the used CHI-AuNP (Fig. 12c and d) sensing surface compared to that of the used CHI-AuNP-TSC (Fig. 12a and b), probably due to the poor adhesion of CHI-AuNP on ITO surface which could detach some AuNPs from the electrode surface. Therefore, it can conclude that the CHI-AuNP-TSC sensing surface was not only outperformed but also more stable than the CHI-AuNP surface.

## Conclusions

Systematic development of a simple and inexpensive portable sensor was carried out on ITO conducting substrate and used for the detection of *E. coli* and *E. aurantiacum* bacteria. AuNPs were synthesized by two different approaches: trisodium citrate and chitosan-stabilized AuNPs and chitosan-stabilized AuNPs. Two different types of AuNPs were immobilized on the working electrode of the fabricated sensor surface. From FESEM and HRTEM results, the difference in size, morphology, and formation of AuNPs on the sensor surfaces was witnessed whereby affected the sensing performance. Optical absorption exhibited the narrower in the SPR absorption band for CHI-AuNP-TSC due to smaller size with larger interparticle spacing. XRD analysis further confirmed the smaller crystal size of CHI-AuNP-TSC than CHI-AuNP. FTIR and XPS analysis further yielded the qualitative and quantitative estimation of the synthesized AuNPs. Electrochemical studies demonstrated that the CHI-AuNP-TSC electrode offered a higher current response with lower charge transfer resistance than the CHI-AuNP electrode. CHI-AuNP-TSC sensor achieved the lowest detection limit of 1.07 CFU/mL and 2.41 CFU/mL with higher sensitivity for the detection of *E. coli* and *E. aurantiacum* and showed better selectivity toward *E. coli*. Moreover, the CHI-AuNP-TSC sensor surface was more stable than CHI-AuNP, which could be a good candidate for further development. In future, the presented sensor can be modified with specific recognition elements such as enzymes, antibodies, and aptamers for specific detection and used as a portable biosensor chip for rapid detection of water and food-borne diseases, environmental monitoring, and point-of-care device.

## Supplementary Information

Additional file1 (DOCX 362 KB)

## Data Availability

All data generated or analyzed during this study are included in this article [and its supplementary information file].
