# Supplementary material for: Development of portable sensor for the detection of bacteria: effect of gold nanoparticle size, effective surface area, and interparticle spacing upon sensing interface
Source: Discov Nano. 2023 Mar 18;18(1):45. doi: 10.1186/s11671-023-03826-4 (PMC10214913; doi:10.1186/s11671-023-03826-4)
Supplement: Supplementary file 1 — Additional file1 (DOCX 362 KB) [file 11671_2023_3826_MOESM1_ESM.docx]

Supporting information of

**Development of portable sensor for the detection of bacteria: Effect of gold nanoparticle size, effective surface area and interparticle spacing upon sensing interface**

Khadija Al-Yahmadi^1^, Htet Htet Kyaw^1,*^, Myo Tay Zar Myint^2^, Rahma Al-Mamari^3^, Sergey Dobretsov^3^, Mohammed Al-Abri^1,4,*^

^1^Nanotechnology Research Center, Sultan Qaboos University, P.O. Box 33, Al-Khoud, Muscat 123, Oman

^2^Department of Physics, College of Science, Sultan Qaboos University, P.O Box 36, Al-Khoud, Muscat 123, Oman

^3^UNESCO Chair. Department of Marine Science and Fisheries, College of Agricultural & Marine Sciences, Sultan Qaboos University, PO Box 34, Al-Khoudh, Muscat 123, Oman

^4^Department of Petroleum and Chemical Engineering, College of Engineering, Sultan Qaboos University, P.O. Box 33, Al Khould, Muscat 123, Oman

^*^Corresponding author: [htet@squ.edu.om](mailto:htet@squ.edu.om); [alabri@squ.edu.om](mailto:alabri@squ.edu.om)

Electrode fabrication process

The details of the electrode fabrication processes are as follows. Firstly, electrode patterns were design using computer software then pattern transfer process was carried out by the following steps. ITO substrates were cut the required dimensions (as mentioned above) and covered the conducting side with vinyl sticker. Subsequently, printed sensor electrode design was placed on vinyl sticker and removed the un-wanted parts by cutting manually. Then, etching process was carried out using 9 M concentrated hydrochloric acid (HCl) at room temperature under constant stirring and the estimated etching rate was ca. 4 Å/s. The etching process was carried out for 15 minutes (commercial ITO has the thickness of around 120 – 150 nm) in order to achieve the complete removal of unwanted ITO area. The optical photos of the EC sensor are shown below.

**(b)**


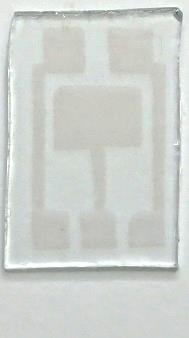


**1 cm**


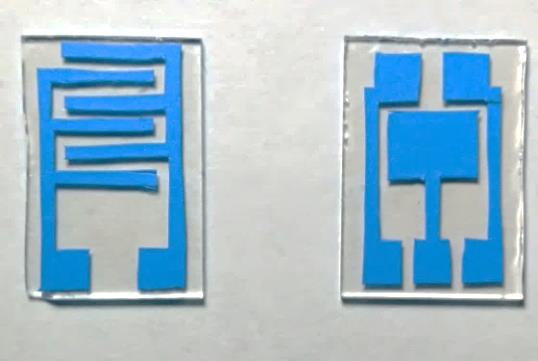


**1 cm**

**WE**

**CE**

**RE**

**(c)**

**(a)**

**Figure S1**. (a) Mask design for EC sensor (b) Optical photo of the ITO substrate being masked with vinyl sticker (c) Optical image of the fabricated three electrodes EC sensor


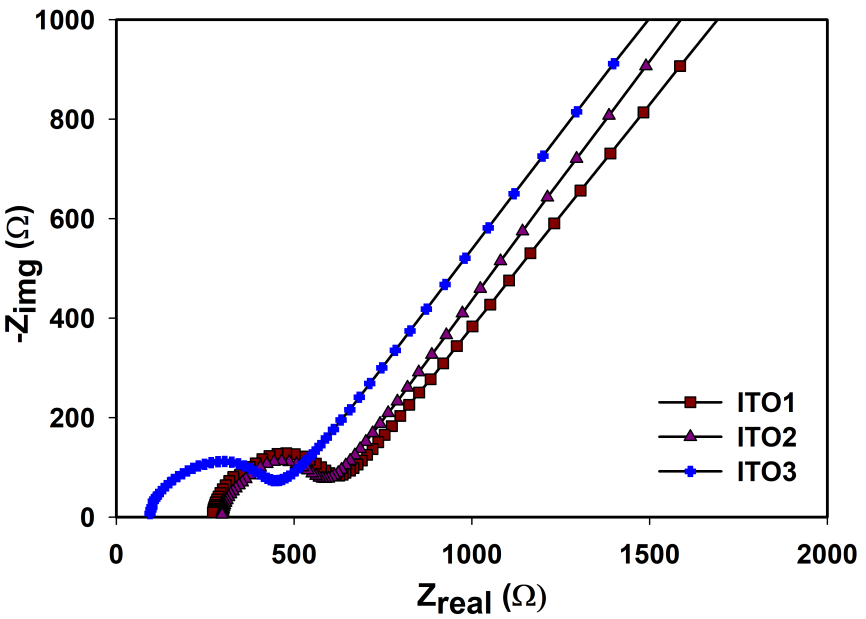


**Figure S2.** Nyquist plots of three bare ITO sensor electrodes


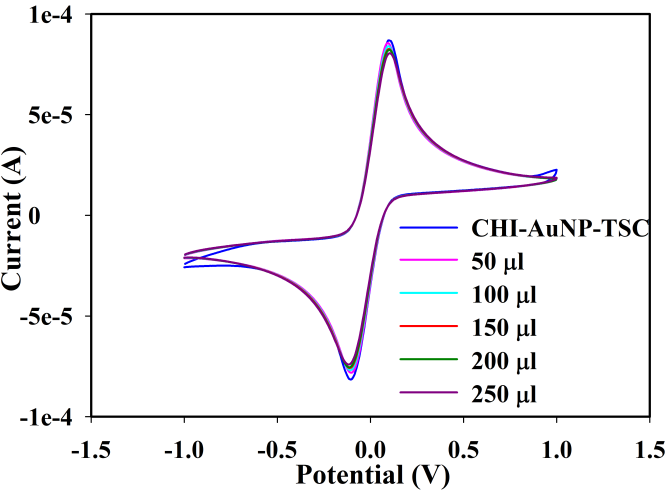

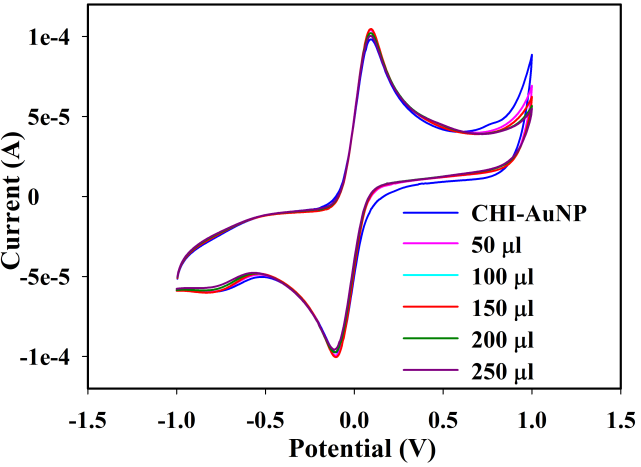


(a)

(b)

**Figure S3.** CV performances of (a) CHI-AuNP-TSC and (b) CHI-AuNP sensing electrodes at different volume of the nutrient broth

Estimation of AuNPs surface area

The surface area of AuNPs on the sensor surface was estimated using FESEM images. Firstly, average particle size was estimated from the particle size distribution graph (Fig. 3a and b) and taken as AuNPs diameter. The total number of particles per unit area was manually counted from the FESEM image which was taken in the same magnification for both of the sample surfaces. Then, the total surface area was calculated by assuming a spherical shape particle. The estimated surface area of CHI-AuNP and CHI-AuNP-TSC was estimated to be ~ 5.57x10^-13^ m^2^ and 6.26x10^-13^ m^2^, respectively, where the original surface was 4.8 x10^-13^ m^2^. Therefore, the surface area increment of 16% for CHI-AuNP and 30% for CHI-AuNP-TSC was witnessed.
